# Size-Specific Copper Nanoparticle Cytotoxicity Varies between Human Cell Lines

**DOI:** 10.3390/ijms22041548

**Published:** 2021-02-04

**Authors:** Ina Na, David C. Kennedy

**Affiliations:** Metrology, National Research Council Canada, 1200 Montreal Road, Ottawa, ON K1A 0R6, Canada; inana96@gmail.com

**Keywords:** copper nanoparticle, cytotoxicity, uptake, stability, spectroscopy

## Abstract

Commercially available copper nanoparticles of three different sizes were tested for cytotoxicity against three human cell lines using four different cytotoxicity assays. This array of data was designed to elucidate trends in particle stability, uptake, and cytotoxicity. The copper nanoparticles are not stable in cell culture media, and rapid changes over the time course of the assays play a critical role in the measured endpoints. Typically, the 40–60 nm particles tested were more cytotoxic than either smaller or larger particles. These particles were also taken up more readily by cells and exhibited different stability dynamics in cell culture media. This provides a good correlation between total cellular uptake of copper and cytotoxicity that may be directly linked to particle stability, though it is unclear why the intermediate-sized particles exhibited these unique properties when compared with both larger and smaller particles.

## 1. Introduction

Copper is an important trace element in humans and is tightly regulated in the body. High levels of copper are maintained in the brain for neurotransmitter production, and in the body, copper is involved in metabolic activity and immune response [1,2]. Diseases associated with both copper excess (e.g., Wilson’s disease [3]) and deficiency (e.g., Menkes disease [4]) and changes in copper homeostasis are also linked to several neurodegenerative disorders, including Alzheimer’s disease, Parkinson’s disease, prion disorders, and Huntington’s disease [5,6,7,8,9,10,11]. With nanoparticles being increasingly incorporated into commercial products, it is important to determine if either the materials or their metabolites in the human body can pose a threat to human health. Copper nanoparticles are used as both drugs to treat osteoporosis [12] and as antibacterial and antifungal agents [13,14,15,16,17,18]. There is also significant interest in using nano-copper as an antiviral material that can be incorporated into personal protective gear and other healthcare products to help prevent the spread of infectious diseases [19]. Nano-copper is also used in large quantities in pesticides [20,21] and as a nutritional supplement in both cattle and poultry feed [22,23]. This intersection with the food chain poses a potential exposure hazard to humans, and direct medical intervention using nano-copper can deliver copper nanoparticles directly into the blood, circumventing defence barriers through other traditional exposure routes such as ingestion and inhalation. While there are studies on copper nanoparticle exposure in animal models [24,25], relatively little is known about their direct effects on human cells and whether or not specific particle sizes pose specific risks, as this size dependence is observed for other particles, such as silver nanoparticles [26,27], another common antimicrobial metal nanoparticle. Further, attempts have been made to create stable suspensions of copper nanoparticles in various aqueous media; however, monitoring the fate of the suspended particles when introduced to cell culture media over incubation periods for cell-based assays remains a challenge. Before in vivo studies can be performed, more in vitro data are needed to determine how the particles may transform in biological matrices and if specific nanoforms pose a heightened risk for cellular cytotoxicity. In this study, we examine the cytotoxic behavior of three sizes of copper nanoparticles against three human cell lines using four different assays to build up an array of biological data correlated with the physical characterization of the particles to examine whether there is a significant risk posed by such particles in the cell lines examined, and, more broadly, whether these in vitro assays suggest that further study in more complex model systems related to exposure through food-based or medical products is needed.

## 2. Results and Discussion

### 2.1. Copper Nanoparticle (CuNP) Characterization in Cell Culture Media

**Figure 1:**
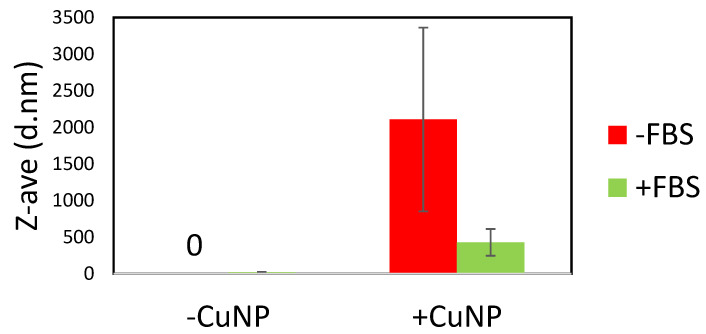
The Z-average of a 10 min probe-sonicated 20 µg/mL suspension of 40–60 nm copper nanoparticles (CuNPs) in media with and without FBS. The conditions were replicated in media without CuNPs as a control.

**Figure 2:**
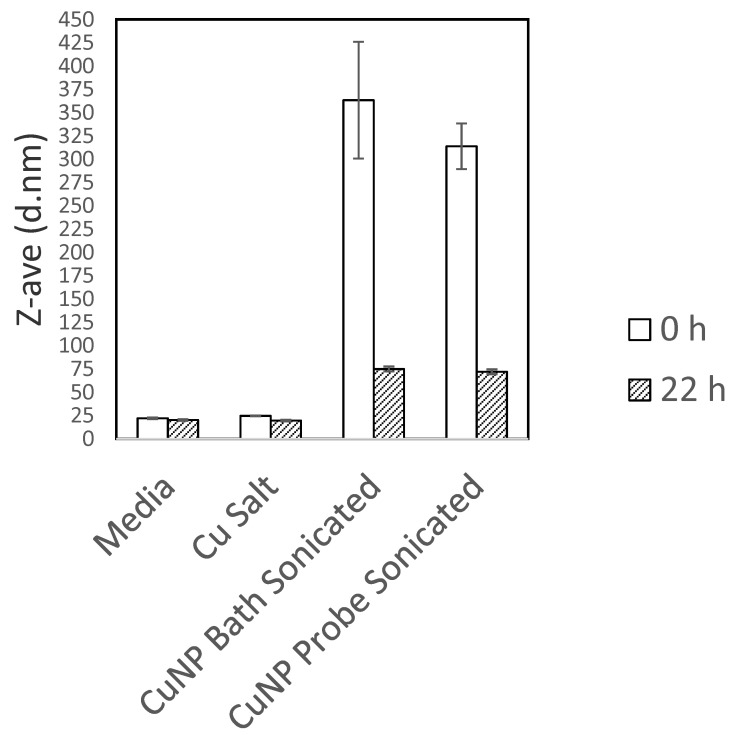
Comparison of the Z-average between complete media, a CuCl_2_ solution in media 20 µg Cu/mL, a bath-sonicated 20 µg/mL 40–60 nm CuNP suspension, and a probe-sonicated 20 µg/mL 40–60 nm CuNP suspension immediately after and 24 h after sonication. Ten minutes of sonication was used each time.

**Figure 3:**
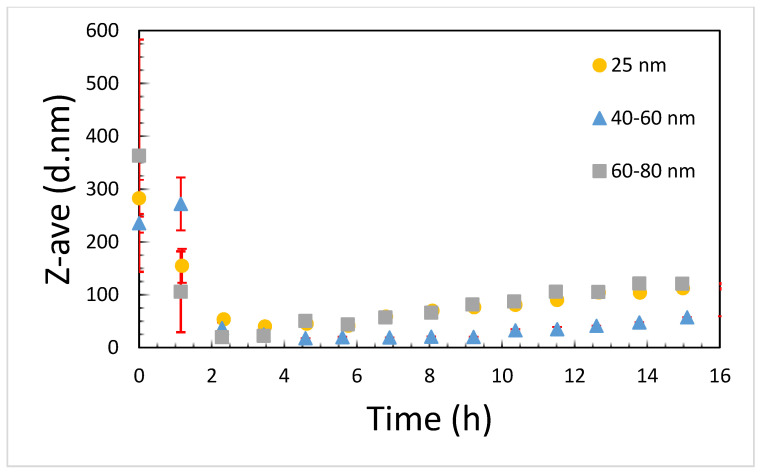
The Z-average diameter of 10 min bath-sonicated 20 µg/mL CuNP suspensions in media over a 16 h period with measurements made approximately every hour. After 16 h, no changes were observed out to 48 h.

Optimization of the copper suspensions to be used for cell culture testing was performed on the copper 40–60 nm sample. We were unable to produce stable dispersions of the material in water or PBS with either bath or probe sonication and the addition of stabilizing polymers such as PVP or PEG did not help to produce stable suspensions of these powder samples. We then decided to disperse the particles directly in media. Sonication can damage proteins in serum, and so we decided to test if the presence of serum affected the dispersability of the particles in a cell culture medium or whether the serum could be added after the particles were dispersed. Figure 1 shows a comparison between particles with and without serum in the media. There is a very clear improvement in the dispersability of the particles when serum is present. We then sought to determine if there was a difference between probe and bath sonication methods. In Figure 2, it is shown that there was no significant difference between the two sonication methods. We also noted that the age of the particle dispersions was critically important. Fresh suspensions had to be prepared immediately prior to the start of each experiment (Figure 2). Particle agglomerates appear to rapidly break down over the first 3 h in media, suggesting that the particles are either dissolving, precipitating, or a combination of the two (Figure 3). Then, over the following 13 h there is a slow increase in the observed hydrodynamic radius, suggesting that either particles are reforming in media, or that precipitated particle agglomerates are breaking down and the individual particles are resuspending. It is also possible that the particles that are reforming may not be copper particles, but rather protein particles agglomerated together by copper ions or small copper complexes. This is observed in human biology, for example, with beta amyloid, where copper ions help drive the oligimerization of protein, resulting in amyloid plaques [11]. There was also no precipitation of the particles observable in the cuvette and sonication of the aged particle–media mixture did not result in a resuspension of any larger particles or agglomerates. While the 25 nm and 60–80 nm particles both end with average hydrodynamic diameters that are approximately 140 nm, the 40–60 nm particles end up measuring less than half that or approximately 60 nm in diameter. The smaller size for the 40–60 nm particles likely is the result of preferential reactions with serum components that promote a more stable suspension, at least at this concentration. It is likely that the stability curves will vary with changes in either particle or serum concentration.

**Figure 4:**
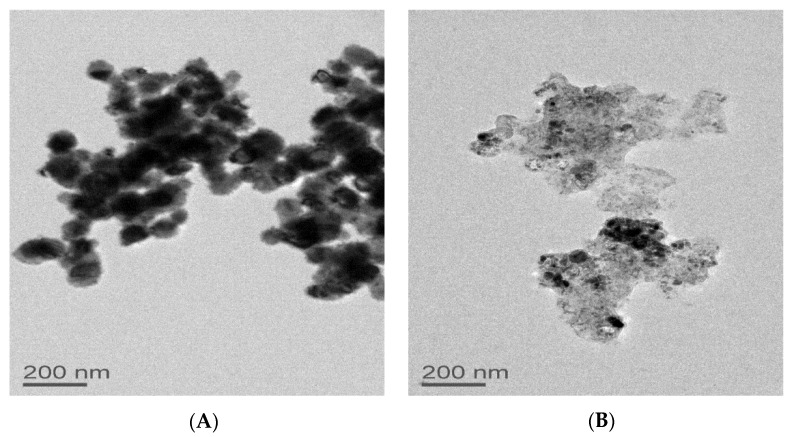
TEM images of 25 nm particles suspended in cell culture media imaged immediately after particle suspension (**A**) and the same sample imaged after 24 h (**B**).

The dissolution of particles from larger aggregates appears to be confirmed by TEM measurements as well. TEM images of the copper particles were measured upon suspension on media with FBS and after 24 h (Figure 4). While the images only show large agglomerates of particles, it appears that these agglomerates lose mass over time, leaving only a biomolecule shell after 24 h, with the copper particles having dissolved from or migrated out of the agglomerates. TEM images of both the 40–60 and 60–80 nm particles in media also only show large agglomerates where individual particles could not be resolved from the larger media–particle agglomerates (see supporting information).

### 2.2. Cytotoxicity of CuNPs

With copper nanoparticles dispersed in complete media, we then measured the cytotoxicity of the particle suspensions against three different cell lines using four different assays. As noted in the previous section, the particles likely dissolved over the time course of the experiment, so attempts were made to try and comprehend if the particles or the dissolved ions were responsible for the observed cytotoxicity. We also noted that at the highest concentrations of copper nanoparticles, large agglomerates could be seen settling on the bottom of the wells; however, after 24 h, these agglomerates were no longer visible and had likely dissolved as was observed in the dynamic light scattering (DLS) samples (see supporting information, Figures S1 and S2).

**Figure 5:**
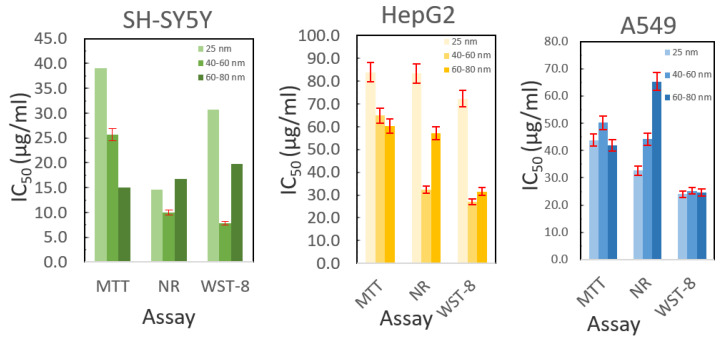
Cytotoxicity data for copper nanoparticles compared using three assays in three cell lines. Error bars are calculated as the standard error from three repetitions, each containing six replicates for each measurement.

Figure 5 summarizes the results from the cytotoxicity assays. For A549 cells, there is no size dependence for the cytotoxicity using the MTT or WST-8 assays, though the magnitude of the measured result differs significantly between these two assays. The neutral red assay does show a size dependency with smaller particles being more cytotoxic. In HepG2 cells, these trends differ greatly. For the MTT assay, there is a gradual trend showing that larger particles are more cytotoxic, while for the other two assays, the greatest cytotoxicity is observed for the 40–60 nm particles (the particles that had the smallest hydrodynamic radius by DLS), with the 25 nm particles being significantly less cytotoxic (a greater hydrodynamic radius by DLS). For the SH-SY5Y cells, the trends are similar to those observed for HepG2 cells. Again, the MTT assay exhibits a size-dependent trend with smaller particles being less cytotoxic, while the other two assays exhibit the highest cytotoxicity for the 40–60 nm particles. From this data, it is inconclusive if there is a size dependency for the particles on cytotoxicity. It is possible that the larger particles settle out of suspension, resulting in higher cytotoxicity in both HepG2 and SH-SY5Y cells as the concentration of particles at the cell surface would be greater. Performing cell-based assays is a constant challenge when nanoparticle suspensions are not stable over the time course of the assay. The higher cytotoxicity observed in some assays for the 40–60 nm particles may be a result of the smaller size observed by DLS. These particles appear to be better dispersed in cell culture media over the time course of these assays and would likely be more bioavailable. Further experiments are needed to determine which of these hypotheses are correct. Rapid dissolution kinetics that could arise over the experimental time course and the different mechanisms the different cell lines have for processing copper ions and copper complexes that form in media may also contribute to the different results. It is not surprising that the assays do not agree as the neutral red assay measures membrane integrity while the MTT and WST-8 assays measure metabolic activity. A broader set of assays is needed along with more time points to determine if there is a size-dependent effect and whether the cytotoxicity values change over time.

**Figure 6:**
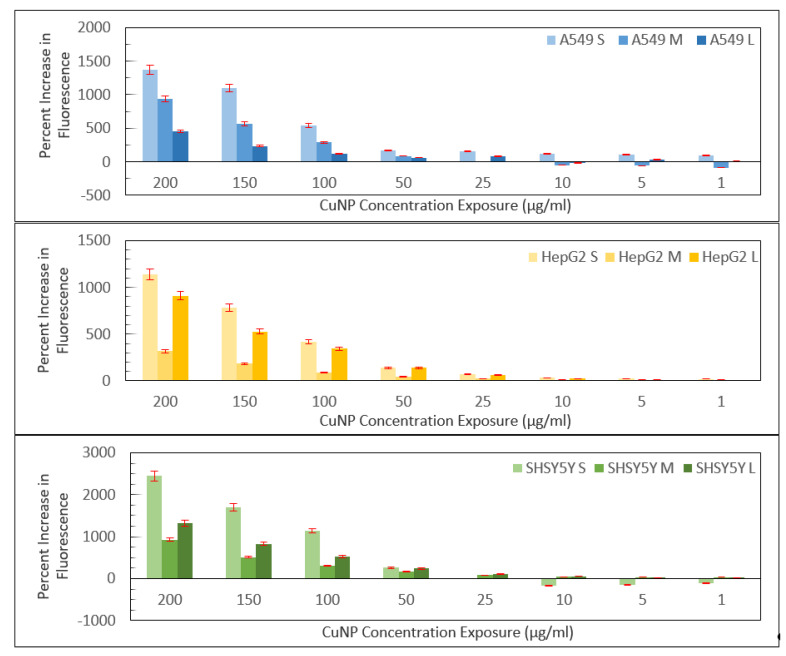
DCFDA results measured for all three nanoparticle sizes in three different cell lines across eight concentrations. Percent increase calculated by calculating the percent increase of fluorescence relative to 0 µg/mL on a plate at 0 h (right after applying the dilution) and after 1 h of incubation and then subtracting the percent increase at 1 h and 0 h.

We next examined the production of reactive oxygen species (ROS) in media (Figure 6). Using the DCFDA assay, we measured the production of ROS at each concentration. Here, we see some interesting correlations with the cytotoxicity data. In both HepG2 and SH-SY5Y cells, the lowest ROS production occurs for the 40–60 nm particles with the ROS production increasing in a dose-dependent manner. These particles are also the most cytotoxic in the WST-8 and neutral red assays, suggesting that the MTT assay may not be an effective assay for accurately measuring the cytotoxicity of CuNPs and that higher levels of ROS production for the smaller and larger nanoparticles correlate with lower cytotoxicity in these cell lines. For the A549 cells, however, there appears to be a correlation between greater ROS production and increased cytotoxicity, as measured by the neutral red assay, with smaller particles being more cytotoxic. For this cell line, neither the MTT nor WST-8 assays showed any size-dependent toxicity. Both of these assays are mitochondrial activity assays and, thus, both may not be appropriate for measuring the cytotoxicity of CuNPs in this cell line as dissolved copper ions may disrupt the assay in a non-toxic manner, making the neutral red assay a superior assay for cytotoxicity measurements. To truly assess if there is a size-dependent link, and how it correlates to ROS production, a greater number of cell lines still need to be tested to establish more consistent trends in the data.

### 2.3. Uptake of CuNPs by Cells

We next sought to determine if the changes in cytotoxicity data with media could be correlated to changes in particle uptake. There is a general trend in uptake between cell lines (Table 1) with HepG2 cells taking up twice as much copper as A549 cells, and about 3 times as much as SH-SY5Y cells. We have previously reported that SH-SY5Y cells take up less silver than other cell lines as well, so this appears to be a consistent observation for these cells with other nanoparticles as well [26]. For all three cell lines, the highest uptake occurs for the 40–60 nm particles; again, the particles with the smallest hydrodynamic radius. In at least two of the cell lines, these particles were also the most cytotoxic and suggest that this intermediate-sized particle is uniquely able to be taken up into the cells more readily than smaller or larger particles, resulting in more acute cytotoxicity, at least in the HepG2 and SH-SY5Y cells. These particles are also observed to be smaller by DLS in cell culture media than either the 25 nm or 60–80 nm particles over the time course of the experiment, suggesting that this size of particle forms smaller agglomerates in this cell culture media, improving its bioavailability. We also performed live cell imaging of CuNPs in A549 cells. Here, the large particle agglomerates could be seen being taken up by cells rapidly in the first 24 h.

## 3. Methods and Materials

### 3.1. Materials

Copper nanoparticles were purchased from Skyspring. Nanomaterials (Houston, TX, USA) as dry black powders were in three sizes—25 nm, 40–60 nm, and 60–80 nm. Image analysis of TEM images of the particles suspended in water validated the sizes reported by the manufacturer and agreed with TEM images provided with the materials. These reported particle sizes were used to describe the samples prepared using these particles in cell culture media, thought DLS measurements of these samples exhibited a high degree of agglomeration. The particles are spherical and without surface coating or stabilizing agents and are greater than 99.8% copper. CuCl_2_ was purchased from Sigma-Aldrich (Oakville, ON, Canada).

### 3.2. Cell Culture

SH-SY5Y (brain), A549 (lung), and HepG2 (liver) cells (American Tissue Culture Center, Manassas, VA, USA) were all grown in Dulbecco’s modified Eagle’s medium (DMEM) (Gibco) supplemented with 10% fetal bovine serum (FBS) (Gibco, Ottawa, ON, Canada) and 1% penicillin–streptomycin (Pen/strep) (50 µg/mL, Gibco) under standard culture conditions (37 °C, 5% CO_2_). Cells were grown in T75 flasks (Falcon, Ottawa, ON, Canada) and Trypsin–EDTA solution (Gibco) was used for passaging cells (3 mL per T75 flask for HepG2 and A549 cells and 2 mL for SH-SY5Y). For passaging, SH-SY5Y cells were treated with Trypsin–EDTA at room temperature for 5 min, while the other two cell lines were incubated for 10 min at 37 degrees.

### 3.3. Nanoparticle Suspension Preparation

Stock suspensions of each nanoparticle size were prepared in clear complete media at 200 μg/mL and bath sonicated for 10 min at 25 °C to disperse the particles. The suspensions were promptly diluted in clear complete media to 150, 100, 50, 25, 10, 5, and 1 µg/mL for immediate use to cover a range of concentrations typically tested for metal and metal-oxide nanoparticle cytotoxicity. Stock samples used for the cytotoxicity experiments for the nanoparticles measured by DLS had the following values in cell culture media with serum immediately after preparation—the 25 nm particles had a hydrodynamic radius of 280 ± 40 with a poldispersity index (PDI) value of 0.36; the 40–60 nm particles had a hydrodynamic radius of 260 ± 20 with a PDI value of 0.22; and the 60–80 nm had a hydrodynamic radius of 390 ± 40 with a PDI value of 0.42. The values changed rapidly upon sample preparation; however, all samples were measured immediately after preparation and dilution in order to minimize errors that could arise if the time between preparation and measurement was different between samples.

### 3.4. Dynamic Light Scattering

Samples were run on a Malvern Zetasizer Nano-ZS. Samples were run in plastic cuvettes (BRAND) with a 1 mL sample volume. Each sample was measured 3 times. Nanoparticles were suspended in water, media without FBS, and complete media using bath and probe sonication methods for dispersing particles at a concentration of 200 μg/mL and were diluted with media as needed to improve the readings.

### 3.5. Transmission Electron Microscopy

Sample preparation: Samples were prepared in the same manner as for DLS at a concentration of 200 μg/mL. Carbon-film-supported TEM grids were glow-discharged. About an 8 µL droplet of CuNP suspension was placed on a TEM grid. After one minute, excess liquid on the grid was blotted away with filter paper and grids were rinsed with deionized water two times to remove salt. TEM grids with CuNPs were then dried in ambient air before being analyzed in the TEM.

Image acquisition: Bright field (BF) SEM images of CuNPs were carried out on a Hitachi S5500 SEM. Individual particles were not observed. Particle analysis of the resulting agglomerates was not possible given the resolution of the images.

### 3.6. (3-(4,5-dimethylthiazol-2-yl)-2,5-diphenyltetrazolium Bromide (MTT) Assay

Cells were seeded into wells in a 96-well plate (Falcon) (1.5 × 105 cells/mL (A549 and HepG2) and 2 × 105 cells/mL (SH-SY5Y), 100 µL per well) to cover a 9 × 6 grid, filling 54 wells. Remaining wells were filled with 200 µL of PBS. After 24 h, the media were removed and 200 µL of each dilution of particles in complete media spanning from 200 µg/mL to 1 µg/mL was added to the seeded wells. For each nanoparticle, eight dilutions were prepared and for each dilution six replicates were performed. In the remaining 6 wells, 100 µL of media was added as a particle-free control. Cells were then incubated with nanoparticles for 24 h. After 24 h, 50 µL of a PBS solution of MTT (2.5 mg/mL) was added to each well and then incubated for 3 h. After 3 h, media were aspirated from all wells, leaving purple formazan crystals in those wells with viable cells. To each well, 150 µL of DMSO was added. Plates were then agitated for 30 s to dissolve the crystals and analyzed using a plate reader (Fluorstar Omega, BMG Labtech.) to determine the absorbance of each well at 570 nm. This reading divided by the average from the reading of the six control wells was plotted to determine the half maximal inhibitory concentration (IC_50_) value of each compound for each cell line. Six replicates were performed for each sample on each cell line for each experiment, and each experiment was repeated three times. The values and errors reported were calculated from 18 unique measurements after curves were fit with a 4-variable sigmoidal curve to calculate the IC_50_ values.

### 3.7. Neutral Red Assay

This procedure was modified from a published protocol [28]. Cells were prepared in a manner identical to that for the MTT assay and treated for 24 h with nanoparticles in a 96-well plate. Neutral red media were prepared as reported. After 24 h, media were aspirated and 100 μL of neutral red media was added to each well. Plates were then incubated for 2 h, at which time the neutral red media were aspirated from the wells. Cells were then washed with 150 μL of PBS, and then 150 μL of destain solution (50% ethanol, 49% water, 1% acetic acid) was added to each well. Plates were then shaken for 10 min to extract the neutral red from the cells and then the absorbance of each well was measured at 540 nm.

### 3.8. Water-Soluble Tetrazolium 8 (WST-8) Assay

Assays were performed using a CCK-8 kit purchased from VitaScientific. Their procedure was adapted to our 24 h protocol used for the MTT assay. After 24 h of treatment, 10 μL of WST-8 reagent was added to each well. Plates were then incubated for 4 h, after which plates were measured at 450 nm.

### 3.9. 2′,7′-dichlorofluorescin Diacetate (DCFDA) Assay

DCFDA assays were performed using abcam ab113851 kits. Cells were prepared in a manner identical to that for the MTT assay in black-walled 96-well plates. Immediately prior to use, DCFDA buffer and solution were prepared as per the assay kit protocol. After seeding the cells overnight, the wells were washed with 100 µL of DCFDA buffer. The wells were then filled with 100 µL of DCFDA solution and incubated in standard culture conditions for 45 min. After incubation, the DCFDA solution was removed and replaced with 100 µL of 1× PBS. The fluorescence was read at Ex/Em 485/535 using a spectrophotometer. The buffer was then removed and replaced with 100 µL of each dilution (200, 150, 100, 50, 25, 10, 5, and 1 µg/mL) with the highest on the left and the lowest on the right side of the plate. The leftmost and rightmost column were filled with 100 µL of complete media. Fluorescence measurements were taken again at Ex/Em = 485/535 nm immediately after the dilutions were applied, after 1, 2, 3, and 24 h. Between each time the fluorescence was measured, the plates were incubated under standard culture conditions.

### 3.10. Metal Analysis

To determine the CuNP uptake into cells, 5 mL cell suspensions of 105 cells/mL of cells were plated into 3 cm Petri dishes. After 24 h, 250 μL of nanoparticles (stock suspensions of 20 μg/mL) was added to the cells. These samples were incubated for 24 h, at which time the media were removed and the cells rinsed twice with PBS. Trypsin–EDTA (2 mL of 0.25%) was then added to detach the cells from the plate surface, and an additional 3 mL of PBS was added to resuspend the cells. These suspensions were transferred to 15 mL conical Falcon tubes and centrifuged for 5 min at 800 rpm. The supernatant was discarded and the cells resuspended and rinsed twice with PBS in this manner to remove particles from the cell surface. Cell pellets were then resuspended in 2 mL of PBS and counted using a LUNA automated cell counter (Logos Biosystems). Cell suspension ranged between 1 and 2 × 106 cells per sample for HepG2 and A549 cells and between 0.5 and 1.5 × 106 cells per sample for SH-SY5Y cells. After counting the cells in each sample, the cells were centrifuged again for 5 min at 2000 rpm and the supernatant discarded. The cell pellet was dried overnight. To each dried pellet, 100 µL of concentrated nitric acid was added and the sample left for 24 h to be digested. Samples were then diluted with H2O and submitted for ICP-MS (Element XR, Thermo Fisher Scientific, Bremen, Germany) analysis to determine the copper content. The results were then normalized to the number of cells in each sample. Each experiment was repeated 3 times and the values and errors reported are the average of these 3 measurements.

## 4. Conclusions

Copper nanoparticles are useful chemical additives to many products due, in large part, to their antimicrobial activity. Here, we have shown that while all copper nanoparticles tested exhibit cytotoxicity against all three cell lines tested, particles in the 40–60 nm range appear to affect the cells more acutely as a result of a smaller hydrodynamic radius in cell culture media and thus efforts to use smaller or larger particles may alleviate possible cytotoxic concerns by forming larger agglomerates in cell culture media. The particle suspensions rapidly change in media over time and may form large agglomerates of protein with copper particles or ions. Further, we determined that while several assays can be used to monitor the cytotoxicity of CuNPs, the neutral red assay appears to be superior to the mitochondrial activity assays—MTT and WST-8. While there is a concentration-dependent increase in fluorescence in the DCFDA assay, the correlation with cytotoxicity between particles of different sizes is inconsistent between cell lines; however, the 40–60 nm particles do appear to cause less oxidative stress yet are also more cytotoxic in two of the cell lines in contrast to the positive relationship in A549 cells, where smaller 25 nm particles are both more cytotoxic and produce more oxidative stress. The 40–60 nm particles are also more readily taken up by cells, suggesting that their smaller size by DLS in cell culture media results in greater uptake into cells. More data on more cell lines are required to determine if ROS are a causative factor in the cytotoxicity of these particles.

## Figures and Tables

**Table 1:** Copper uptake analysis was performed on cell pellets treated with CuNPs for 24 h in cell culture media. Values reported are in ng copper/10^6^ cells with calculated standard errors from three repeated measurements.

Cell Line	25 nm CuNPs	40–60 nm CuNPs	60–80 nm CuNPs
A549	250 ± 20	400 ± 10	250 ± 20
HepG2	450 ± 20	750 ± 40	600 ± 40
SH-SY5Y	140 ± 20	200 ± 20	130 ± 20

## Data Availability

The data presented in this study are available on request from the corresponding author.

## Supplementary Materials

Supplementary materials can be found at https://www.mdpi.com/1422-0067/22/4/1548/s1.

## Abbreviations

CuNP	copper nanoparticle
DCFDA	2′,7′-dichlorofluorescin diacetate
MTT	(3-(4,5-dimethylthiazol-2-yl)-2,5-diphenyltetrazolium bromide
WST	Water-Soluble Tetrazolium
IC_50_	the half maximal inhibitory concentration
ROS	reactive oxygen species
FBS	fetal bovine serum
TEM	transmission electron microscopy
