# Supplementary material for: Size-Specific Copper Nanoparticle Cytotoxicity Varies between Human Cell Lines
Source: Int J Mol Sci. 2021 Feb 4;22(4):1548. doi: 10.3390/ijms22041548 (PMC7913709; doi:10.3390/ijms22041548)

Supporting Information

Size specific copper nanoparticle cytotoxicity varies between human cell lines

Ina Na, and David C. Kennedy*

Metrology, National Research Council Canada, 1200 Montreal Road, Ottawa K1A 0R6, Canada;

*david.kennedy@nrc-cnrc.gc.ca

Figure S1. TEM images for copper nanoparticles in media supplemented with fetal bovine serum

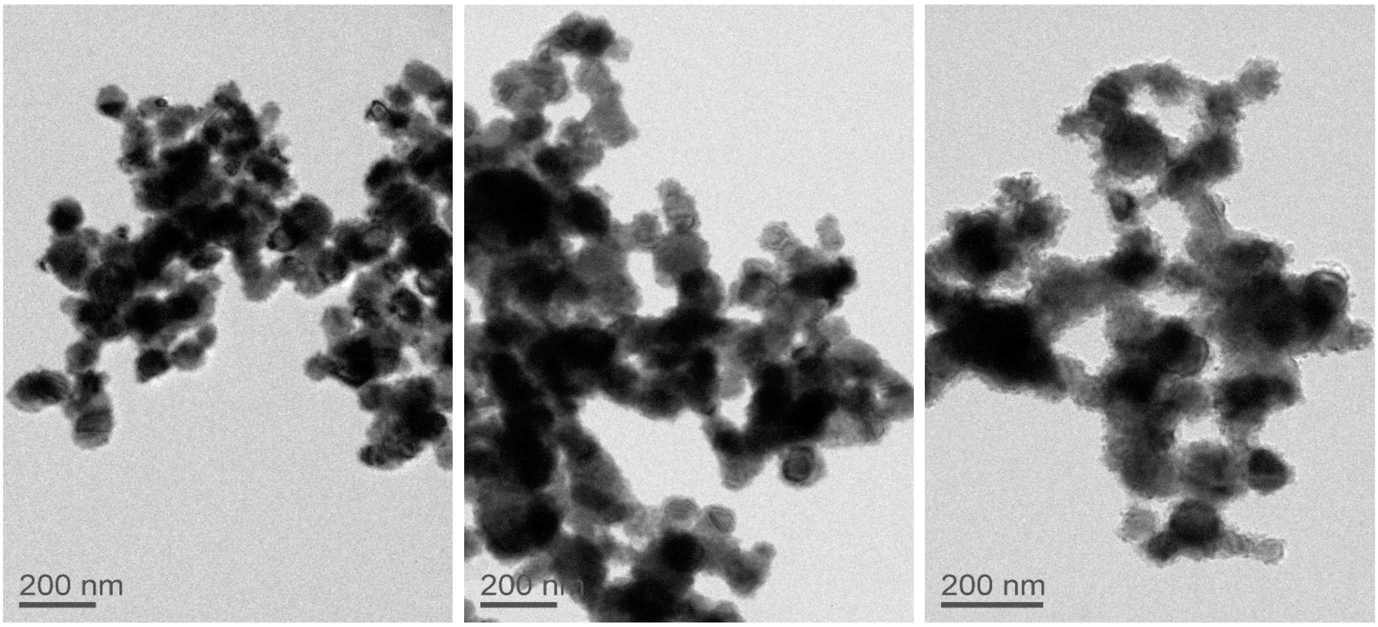

25 nm CuNPs 40-60 nm CuNPs 60-80 nm CuNPs

Figure S2. Brightfield images of A549 (left), HepG2 (middle) and SH-SY5Y (right) cells exposed to 200 µg/mL of 25 nm CuNPs immediately after exposure. Large black CuNP agglomerates are easily observed but are not present after 24 h exposure.

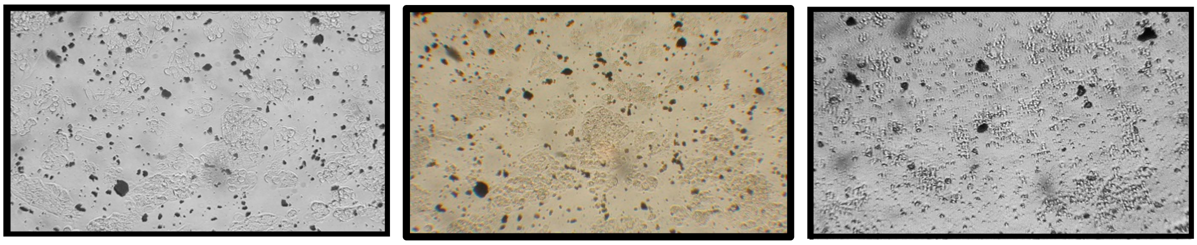

Supplement: Supplementary file 1 [file ijms-22-01548-s001.pdf]
